# OP61 Target Trial Emulation To Determine The Population-Level Cost-Effectiveness Of Multigene Panel Sequencing For Advanced Melanoma

**DOI:** 10.1017/S0266462324001211

**Published:** 2025-01-07

**Authors:** Emanuel Krebs, Deirdre Weymann, Samantha Pollard, Dean Regier

## Abstract

**Introduction:**

Compared to single-gene BRAF testing to guide targeted treatment with BRAF/MEK inhibitors for advanced melanoma, multigene panels can identify additional gene mutations with known therapeutic or prognostic relevance. Implementation of multigene panels remains uneven across healthcare systems given an uncertain clinical and economic evidence base. We determined the population-level cost-effectiveness of multigene panels compared to single-gene BRAF testing for advanced melanoma.

**Methods:**

Our population-based retrospective study emulated a hypothetical pragmatic trial comparing multigene panel sequencing to single-gene BRAF testing. We drew on comprehensive patient-level clinical and health administrative data between September 2016 and December 2018 in British Columbia, Canada. To emulate random treatment assignment, we 1:1 matched multigene panel patients to contemporaneous single-gene tested controls using genetic algorithm-based matching. We estimated three-year overall survival and healthcare costs (2021 CAD), and incremental net monetary benefit (INMB) for life years gained (LYG) using inverse probability of censoring weighted linear regression and nonparametric bootstrapping. We also estimated overall survival using Weibull regression and Kaplan–Meier survival analysis.

**Results:**

We matched 147 patients with advanced melanoma receiving multigene panel sequencing to contemporaneous single-gene-tested controls, achieving good balance for all 15 baseline clinical and sociodemographic covariates. After matching, mean incremental costs were CAD19,447 (USD14,217) (95% confidence interval [CI]: −CAD18,517 [−USD13,537], CAD76,006 [USD55,565]; p=0.41) and mean incremental LYG were 0.22 (95% CI: −0.05, 0.49; p=0.12). We found uncertain differences on overall survival using Kaplan–Meier (stratified Log-rank test p=0.11) and Weibull regression (HR: 0.73 [95% CI: 0.51, 1.03]; p=0.07) survival analysis. Cost differences were driven by systemic therapy (∆C: CAD8,665 [USD6,334]; 95% CI: −CAD36,387 [−USD26,600], CAD53,716 [USD39,268]; p=0.71). The INMB at CAD100,000(USD73,104)/LYG was CAD2,646 (USD1,934) (95% CI: −CAD30,044 [−USD21,963], CAD43,416 [USD31,739]; p=0.89), with a 52.8 percent probability of being cost effective.

**Conclusions:**

There were clinically relevant but uncertain differences in improved survival associated with multigene panel sequencing for advanced melanoma, and the cost-effectiveness of panel-based testing was finely balanced. This real-world evidence generated using randomized trial design principles can support jurisdictions’ deliberations on the reimbursement of precision oncology interventions.

